# Active Travel and Mild Cognitive Impairment among Older Adults from Low- and Middle-Income Countries

**DOI:** 10.3390/jcm10061243

**Published:** 2021-03-17

**Authors:** Lee Smith, Nicola Veronese, Guillermo F. López-Sánchez, Lin Yang, Damiano Pizzol, Laurie T. Butler, Yvonne Barnett, Mireia Felez-Nobrega, Louis Jacob, Jae Il Shin, Mark A. Tully, Trish Gorely, Hans Oh, Ai Koyanagi

**Affiliations:** 1 The Cambridge Centre for Sport and Exercise Sciences, Anglia Ruskin University, Cambridge CB1 1PT, UK; lee.smith@aru.ac.uk; 2 Geriatric Unit, Department of Internal Medicine and Geriatrics, University of Palermo, 90133 Palermo, Italy; ilmannato@gmail.com; 3 Vision and Eye Research Institute, School of Medicine, Faculty of Health, Education, Medicine and Social Care, Anglia Ruskin University, Cambridge CB1 1PT, UK; guillermo.lopez-sanchez@aru.ac.uk; 4 Department of Cancer Epidemiology and Prevention Research, Cancer Care Alberta, Alberta Health Services, Calgary, AB T2S 3C3, Canada; lin.yang@ahs.ca; 5 Italian Agency for Development Cooperation, 33 Street, Amarat, Khartoum 79371, Sudan; damianopizzol8@gmail.com; 6 Faculty of Science and Engineering, Anglia Ruskin University, Cambridge CB1 1PT, UK; laurie.butler@aru.ac.uk (L.T.B.); yvonne.barnett@aru.ac.uk (Y.B.); 7 Research and Development Unit, Parc Sanitari Sant Joan de Déu, CIBERSAM, C/Dr.Antoni Pujadas 42, Sant Boi de Llobregat, 08830 Barcelona, Spain; m.felez@pssjd.org (M.F.-N.); louis.jacob.contacts@gmail.com (L.J.); 8 Faculty of Medicine, University of Versailles Saint-Quentin-en-Yvelines, 78180 Montigny-le-Bretonneux, France; 9 Department of Pediatrics, Yonsei University College of Medicine, Yonsei-ro 50, Seodaemun-gu, Seoul 03722, Korea; SHINJI@yuhs.ac; 10 Institute of Mental Health Sciences, School of Health Sciences, Ulster University, Newtownabbey BT37 0QB, UK; m.tully@ulster.ac.uk; 11 Centre for Health Science, Department of Nursing and Midwifery, University of the Highlands and Islands, Inverness IV2 3JH, UK; trish.gorely@uhi.ac.uk; 12 Suzanne Dworak Peck School of Social Work, University of Southern California, Los Angeles, CA 90007, USA; hansoh@usc.edu; 13 ICREA, Pg. Lluis Companys 23, 08010 Barcelona, Spain

**Keywords:** active travel, older adults, low- and middle-income countries, mild cognitive impairment

## Abstract

Active travel may be an easily achievable form of physical activity for older people especially in low- and middle-income countries (LMICs), but there are currently no studies on how this form of physical activity is associated with a preclinical state of dementia known as mild cognitive impairment (MCI). Therefore, we aimed to investigate the association between active travel and MCI among adults aged ≥50 years from six LMICs. Cross-sectional, community-based data from the World Health Organization’s Study on Global Ageing and Adult Health were analyzed. The definition of MCI was based on the National Institute on Ageing-Alzheimer’s Association criteria. Active travel (minutes/week) was assessed with questions of the Global Physical Activity Questionnaire (GPAQ) and presented in tertiles. Multivariable logistic regression analysis was conducted to assess the association between active travel and MCI. Data on 32715 people aged ≥50 years (mean age 62.4 years; 52.1% females) were analyzed. Compared to the highest tertile of active travel, the lowest tertile was associated with 1.33 (95%CI = 1.14–1.54) times higher odds for MCI overall. This association was particularly pronounced among those aged ≥65 years (OR = 1.70; 95%CI = 1.32–2.19) but active travel was not associated with MCI among those aged 50–64 years. In conclusion, low levels of active travel were associated with a significantly higher odds of MCI in adults aged ≥65 years in LMICs. Promoting active travel among people of this age group in LMICs via tailored interventions and/or country-wide infrastructure investment to provide a safe environment for active travel may lead to a reduction in MCI and subsequent dementia.

## 1. Introduction

The World Health Organisation (WHO) defines dementia as a syndrome in which there is deterioration in cognitive function (i.e., the ability to process thought) beyond what might be expected from normal ageing, and this condition may present symptomatically as memory loss, confusion, disorientation, difficulty speaking or understanding language, and other symptoms [1]. Dementia is most prevalent among older adults with its prevalence estimated to be between 5% and 8% among those aged ≥60 years. In addition to the detrimental impact of dementia on an individual’s quality of life and wellbeing, the care and support of patients with dementia has wide-ranging consequences for families, health-care systems, and society as a whole [2]. Since there is no known cure for dementia, interventions to prevent or delay the emergence of this syndrome are essential.

Mild cognitive impairment (MCI) is a preclinical state of dementia with a high conversion rate to dementia (annual conversion rates ranging from 10% to 15% in clinical samples and 3.8% to 6.3% in community-based samples) [3,4,5], and is increasingly being recognized as an important “target” for the prevention of dementia. One potential correlate of MCI is that of physical activity. Indeed, not meeting physical activity recommendations is considered one of the seven main potentially modifiable risk factors for Alzheimer’s Disease (a predominant form of dementia) and explains approximately 13% (nearly 4.3 million) of Alzheimer’s Disease cases worldwide [6]. Furthermore, an evolving body of literature documents significant benefits of long-term, regular physical activity on cognition while it also reduces dementia risk in people with MCI [7,8,9,10,11,12,13].

However, to the best of our knowledge, there are no studies examining the relationship between active travel (i.e., walking and cycling to and/or from a destination) and MCI. Active travel is often performed at a moderate intensity [14] and thus, it is possible that this is an ideal way to ensure physical activity in older adults. Indeed, transport is a necessity of everyday life, whereas leisure physical activity is possibly an additional burden and is difficult to sustain long term. Therefore, encouraging active travel is a feasible approach to increasing levels of moderate physical activity in older adults [15].

Given this, the aim of the present study was to investigate the association between active travel and MCI in a sample of 32,715 people aged ≥50 years from six low- and middle-income countries (LMICs). Focusing on LMICs is important as the majority (approximately 60%) of those with dementia reside in this setting [1], while the individual, economic, and societal burden of dementia is increasing particularly rapidly in LMICs. Furthermore, levels of physical inactivity [16] are high in these countries, while active travel has been reported to be a common form of physical activity especially among older adults in low-and middle-income settings [15].

## 2. Methods

### 2.1. The Survey

Data from the Global Ageing and Adult Health Survey (SAGE) were analyzed. These data are publicly available through http://www.who.int/healthinfo/sage/en/ (accessed on 18 February 2021). This survey was undertaken in China, Ghana, India, Mexico, Russia, and South Africa between 2007 and 2010. These countries broadly represent different geographical locations and levels of socio-economic and demographic transition. Based on the World Bank classification at the time of the survey, Ghana was the only low-income country, and China and India were lower middle-income countries although China became an upper middle-income country in 2010. The remaining countries were upper middle-income countries.

Details of the survey methodology have been published elsewhere [17]. In brief, in order to obtain nationally representative samples, a multistage clustered sampling design method was used. The sample consisted of adults aged ≥18 years with oversampling of those aged ≥50 years. Trained interviewers conducted face-to-face interviews using a standard questionnaire. Standard translation procedures were undertaken to ensure comparability between countries. If a respondent was unable to undertake the interview because of limited cognitive function, then a separate questionnaire was administered to a proxy respondent (e.g., care giver). These individuals were not included in the current study. The survey response rates were: China 93%; Ghana 81%; India 68%; Mexico 53%; Russia 83%; and South Africa 75%. Sampling weights were constructed to adjust for the population structure as reported by the United Nations Statistical Division. Ethical approval was obtained from the World Health Organization Ethical Review Committee and local ethics research review boards. Written informed consent was obtained from all participants.

### 2.2. Active Travel

Active transport was assessed with questions of the Global Physical Activity Questionnaire (GPAQ) [18]. Participants were asked about the usual way to travel to and from places (e.g., getting to work, to shopping, to the market, to place of worship etc). The answers for two questions were used to calculate the minutes spent in active travel per week: (a) In a typical week, on how many days do you walk or bicycle for at least 10 min continuously to get to and from places?; (b) How much time would you spend walking or bicycling for travel on a typical day? Minutes spent in active travel per week was categorized as low (0–40 min/week), moderate (42–210 min/week), and high (≥217 min/week) based on tertiles [15,19].

### 2.3. Mild Cognitive Impairment

MCI was ascertained based on the recommendations of the National Institute on Aging-Alzheimer’s Association [20]. We applied the identical algorithms used in previous SAGE publications to identify MCI [21,22]. Briefly, individuals fulfilling all of the following conditions were considered to have MCI:

(a) Concern about a change in cognition: Individuals who replied ‘bad’ or ‘very bad’ to the question “How would you best describe your memory at present?” and/or those who answered ‘worse’ to the question “Compared to 12 months ago, would you say your memory is now better, the same or worse than it was then?” were considered to have this condition.

(b) Objective evidence of impairment in one or more cognitive domains: was based on a <−1 SD cut-off after adjustment for level of education and age. Cognitive function was assessed through the following performance tests: word list immediate and delayed verbal recall from the Consortium to Establish a Registry for Alzheimer’s Disease [23], which assessed learning and episodic memory; digit span forward and backwards from the Weschler Adult Intelligence Scale [24], that evaluated attention and working memory; and the animal naming task [23], which assessed verbal fluency.

(c) Preservation of independence in functional abilities: was assessed by questions on self-reported difficulties with basic activities of daily living (ADL) in the past 30 days [25]. Specific questions were: “How much difficulty did you have in getting dressed?” and “How much difficulty did you have with eating (including cutting up your food)?” The answer options were none, mild, moderate, severe, and extreme (cannot do). Those who answered either none, mild, or moderate to both of these questions were considered to have preservation of independence in functional activities. All other individuals were deleted from the analysis (935 individuals aged ≥ 50 years).

(d) No dementia: Individuals with a level of cognitive impairment severe enough to preclude the possibility to undertake the survey were not included in the current study.

### 2.4. Control Variables

The control variables were selected based on past literature and included age, sex, wealth quintiles based on income, years of education received, setting (rural or urban), alcohol consumption in the past 30 days, smoking (never, current, past), sleep problems, anxiety, depression, chronic physical conditions (i.e., diabetes, hypertension, stroke), obesity (body mass index ≥ 30 kg/m^2^ based on measured weight and height), and other physical activity (work and leisure) [26,27]. Those having severe or extreme problems with sleeping in the past 30 days, such as falling asleep, waking up frequently during the night or waking up too early in the morning, were considered to have sleep problems [28]. Anxiety was defined as having severe or extreme problems with worry or anxiety in the last 30 days [29]. Questions based on the World Mental Health Survey version of the Composite International Diagnostic Interview [30] were used for the endorsement of past 12-month DSM-IV depression [31]. Diabetes and stroke were based solely on lifetime self-reported diagnosis. Hypertension was defined as having at least one of: systolic blood pressure ≥ 140 mmHg; diastolic blood pressure ≥ 90 mmHg; or self-reported diagnosis. Other physical activity referred to minutes spent per week in moderate-to-vigorous physical activity in relation to work and leisure activities but not active travel. In line with a previous publication using the same dataset, work and leisure physical activity were dichotomized as ≥150 or <150 min/week [19].

### 2.5. Statistical Analysis

All analyses were done with Stata statistical software version 14.2 (Stata Corp LP, College Station, TX, USA). The analysis was restricted to those aged ≥50 years. Middle-aged people were also included in this analysis as cognitive dysfunction can emerge up to 10 years before a dementia diagnosis [32], and there is increasing evidence that intervening in mid-life is important [33,34,35,36]. Multivariable logistic regression analyses were used to estimate the association between levels of active travel in tertiles (exposure) and MCI (outcome). Analyses using the overall sample and age-stratified samples (i.e., 50–64, ≥65 years) were conducted. We also conducted country-wise analysis to assess whether there is between-country heterogeneity in the association between active travel and MCI. This analysis was restricted to those aged ≥65 years and used a dichotomous active travel variable (i.e., low vs. moderate/high) as preliminary analysis showed that the association between active travel was particularly pronounced in this age group while there were no significant differences between moderate and high levels of active travel. Furthermore, we calculated the Higgins’s *I*^2^ based on estimates from each country. The Higgins’s *I*^2^ represents the degree of heterogeneity that is not explained by sampling error with a value of <40% often considered as negligible and 40–60% as moderate heterogeneity [37]. A pooled estimate was obtained by random-effect meta-analysis.

All regression analyses were adjusted for age, sex, wealth, education, setting, alcohol consumption, smoking, sleep problems, anxiety, depression, diabetes, hypertension, stroke, obesity, work physical activity, leisure physical activity, and country with the exception of the country-wise analysis which did not adjust for country. Adjustment for country was conducted by including dummy variables for each country as in previous SAGE publications [38,39]. All variables were included in the models as categorical variables with the exception of age and education (continuous variables). The sample weighting and the complex study design were taken into account in all analyses with Taylor linearization methods to obtain nationally representative estimates. Results from the logistic regression models are presented as odds ratios (ORs) with 95% confidence intervals (95%CIs). The level of statistical significance was set at *p* < 0.05.

## 3. Results

**Figure 1:**
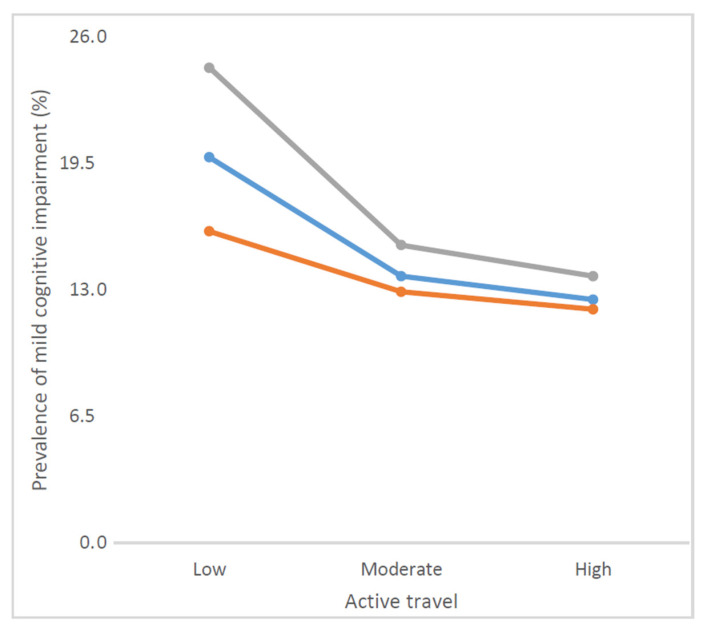
Prevalence of mild cognitive impairment by levels of active travel.

**Figure 2:**
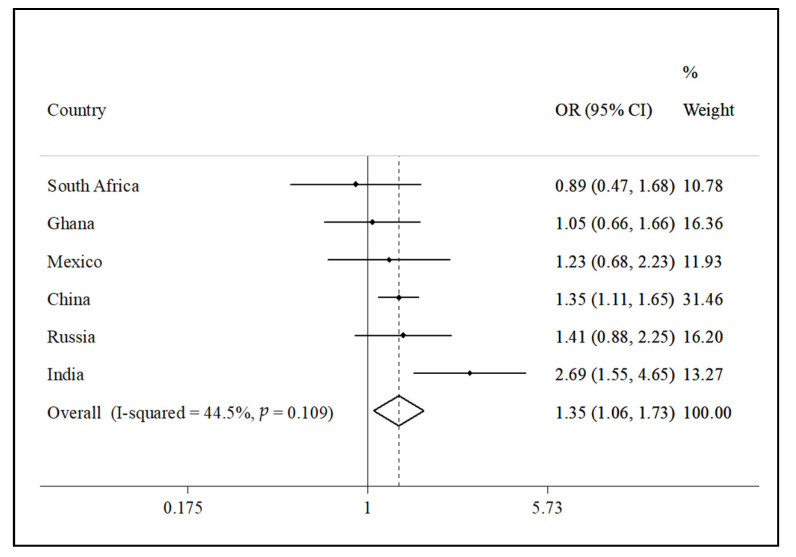
Country-wise association between low active travel (vs. moderate or high active travel) among adults aged ≥65 years estimated by multivariable logistic regression. Abbreviation: OR Odds ratio; CI Confidence interval. Models are adjusted for age, sex, wealth, education, setting, alcohol consumption, smoking, sleep problems, anxiety, depression, diabetes, hypertension, stroke, obesity, work physical activity, and leisure physical activity. Overall estimate was obtained by meta-analysis with random effects.

The final sample consisted of a total of 32,715 people aged ≥50 years with preservation in functional abilities (China *n* = 12,815; Ghana *n* = 4201; India *n* = 6191; Mexico *n* = 2070; Russia *n* = 3766; South Africa *n* = 3672). The sample characteristics are shown in Table 1. The mean age of the sample was 62.4 years and 52.1% were females. The prevalence of MCI was 14.8% overall. The prevalence of <−1 SD for each cognitive performance test and severity of activities of daily living for the overall sample and for those with MCI are shown in Tables S1 and S2, respectively (Supplementary Materials). The median (IQR) of active travel per week was 140 (0–360) min per week. The prevalence of MCI decreased with increasing levels of active travel (Figure 1). For example, overall, the prevalence of MCI was 19.8% among those with low levels of active travel but this decreased to 12.5% among people with high levels of active travel. After adjustment for potential confounders, compared to high levels of active travel, low levels were associated with 1.33 (95%CI = 1.14–1.54) times higher odds for MCI overall (Table 2). The results of the full regression with all covariates are shown in Table S3 (Supplementary Materials) This association was particularly pronounced among those aged ≥65 years (OR = 1.70; 95%CI = 1.32–2.19) but active travel was not associated with MCI among those aged 50–64 years. Country-wise associations between low levels of active travel (vs. moderate or high) among those aged ≥65 years are shown in Figure 2. Low active travel was positively associated with MCI (i.e., OR > 1) in all countries with the exception of South Africa. The overall estimate was OR = 1.35 (95%CI = 1.06–1.73). A moderate level of between-country heterogeneity was observed (*I*^2^ = 44.5%).

## 4. Discussion

### 4.1. Main Findings

In this large sample of older adults from six LMICs, we found that compared to high levels of active travel, low levels were associated with 1.33 times higher odds for MCI. The association was strong in those aged ≥65 years but non-significant among middle-aged adults (50–64 years). Country-wise analysis showed that the association observed has a moderate level of between-country heterogeneity. To the best of our knowledge, this is the first study on active travel and MCI in LMICs.

### 4.2. Interpretation of the Findings

The results of our study are in line with those of previous studies which have found associations between physical activity and various forms of cognitive impairment including dementia [7,8,9,10,11,12,13]. There are several plausible pathways that may explain the association between active travel and MCI. First, participation in physical activity may increase or maintain cognitive reserve, through increased brain perfusion [9]. Second, participation in physical activity via active travel aids in the prevention of non-communicable diseases (e.g., cardiovascular disease and cancer) [40] and certain non-communicable diseases such as cardiovascular disease are associated with cognitive impairment [41]. Finally, active travel requires way-finding and potential reliance on cognitive maps using such cognitive resources may offer some protection against cognitive decline and MCI [42].

It should be noted that in the present study, active travel was associated with MCI in those aged ≥65 years but not those aged 50–64 years (middle-aged). Previous studies have also found that the correlates of MCI differ between the middle-aged and older population [27]. One reason for this discrepancy in findings may be that those aged between 50 and 64 years are at limited risk of MCI in comparison to those aged ≥65 years [43], while they are also less likely to have chronic conditions, and thus, it is possible that the beneficial impact of active travel does not emerge until one reaches the age in which the risk of MCI is high. However, clearly more future research is needed to understand the reason for the age-difference.

### 4.3. Public Health and Policy Implications

Findings from the present study suggest that active travel interventions targeted at those aged ≥65 years in LMICs may aid in the prevention of MCI and ultimately dementia. Older adults can be encouraged to partake in active travel by interventions tailored to their needs, targeted at the most inactive or at those most motivated to change, and delivered either at the level of the individual or household or through group-based approaches [44]. Moreover, at the country-wide level, active travel may be encouraged in older adults through the provision of safe places to actively travel such as well-maintained footpaths and cycle lanes. This is a crucial issue especially in rural settings where the distances between villages are challenging and the pathways are impracticable especially during the rainy season [44].

### 4.4. Strengths and Limitations

The large representative sample of older adults from six LMICs is a clear strength of the present study. Furthermore, standardized methods to assess MCI was used across all countries. However, findings must be interpreted in light of the study’s limitations. First, amount of active travel and some control variables were self-reported potentially introducing self-report and recall bias into the analyses. For example, older adults with MCI may be less likely to accurately recall their active travel. Second, analyses were cross-sectional in design and it is not known whether low levels of active travel precede MCI or whether MCI precedes low levels of active travel. Since people with MCI may be more likely to fall, walk unstably, and get lost, this might have made active travel less possible in these individuals. Third, the control variable “anxiety” was defined as having self-reported severe or extreme problems with worry or anxiety in the last 30 days. The present analysis was thus not able to control for specific types of anxiety (e.g., general anxiety disorder). Fourth, there are currently no standard definitions for MCI especially in population-based studies. We have used a definition used in previous SAGE studies which was based on the National Institute on Aging-Alzheimer’s Association. However, it is possible for the results to have differed if a different definition was used. Relatedly, there is currently no consensus in terms of the acceptable level of functional impairment that individuals with MCI should present [45]. The definition of preservation of independence in functional abilities used in our study was rather conservative. This was done to avoid the omission of MCI cases with disability not related to their cognitive ability. Finally, because the study was not designed to generate clinical diagnoses of dementia, some individuals with mild dementia may have been included in our analytical sample. These individuals may have been classified as MCI and presented with apathy and social withdrawal which are frequent in some dementia subtypes. This in turn could have affected the level of physical activity.

## 5. Conclusions

In this large representative sample of older adults from six LMICs, it was found that low levels of active travel were associated with a significantly higher odds of MCI in those aged ≥65 years. Future research of a longitudinal nature is now required to determine the direction of the association. If confirmed with longitudinal studies, our study results suggest that promotion of active travel among those aged ≥65 years in LMICs via tailored interventions and/or country-wide infrastructure investment to provide a safe environment for active travel may reduce the risk for future MCI/dementia onset.

## Figures and Tables

**Table 1:** Sample characteristics (overall and by age groups).

			Age	
Characteristic		Overall	50–64 Years	≥65 Years
		(*n* = 32,715)	(*n* = 19,092)	(*n* = 13,623)
Mild cognitive impairment	No	85.2	86.7	82.6
	Yes	14.8	13.3	17.4
Active travel	High	31.5	35.2	25.4
	Moderate	35.1	36.3	33.0
	Low	33.4	28.5	41.6
Age (years)		62.4 (0.2)	56.3 (0.1)	72.6 (0.1)
Sex	Male	47.9	49.7	45.0
	Female	52.1	50.3	55.0
Wealth	Poorest	17.1	14.4	21.7
	Poorer	19.0	17.7	21.0
	Middle	19.5	19.0	20.4
	Richer	21.3	23.6	17.5
	Richest	23.1	25.3	19.4
Education (years)		6.0 (0.2)	6.5 (0.2)	5.2 (0.2)
Setting	Rural	53.8	56.6	49.4
	Urban	46.2	43.4	50.6
Alcohol consumption	No	81.3	78.4	86.1
	Yes	18.7	21.6	13.9
Smoking	Never	58.6	56.3	62.2
	Current	34.9	38.3	29.3
	Past	6.6	5.4	8.5
Sleep problems	No	91.3	93.4	87.8
	Yes	8.7	6.6	12.2
Anxiety	No	91.9	92.9	90.3
	Yes	8.1	7.1	9.7
Depression	No	94.0	94.2	93.5
	Yes	6.0	5.8	6.5
Diabetes	No	93.2	94.2	91.4
	Yes	6.8	5.8	8.6
Hypertension	No	45.0	50.0	36.6
	Yes	55.0	50.0	63.4
Stroke	No	97.0	97.9	95.4
	Yes	3.0	2.1	4.6
Obesity	No	88.5	87.9	89.6
	Yes	11.5	12.1	10.4
Work physical activity	≤150 min/week	40.2	33.0	52.7
	>150 min/week	59.8	67.0	47.3
Leisure physical activity	≤150 min/week	89.8	89.2	90.7
	>150 min/week	10.2	10.8	9.3

Data are % or mean (standard error).

**Table 2:** Association between active travel and mild cognitive impairment (outcome) estimated by multivariable logistic regression.

		Overall		Age 50–64 Years		Age ≥65 Years	
		OR	95%CI	OR	95%CI	OR	95%CI
Active travel	High	1.00		1.00		1.00	
	Moderate	0.97	(0.84,1.13)	0.95	(0.79,1.14)	1.04	(0.82,1.31)
	Low	1.33 *	(1.14,1.54)	1.07	(0.89,1.30)	1.70 *	(1.32,2.19)

Abbreviation: OR Odds ratio; CI Confidence interval. Models are adjusted for age, sex, wealth, education, setting, alcohol consumption, smoking, sleep problems, anxiety, depression, diabetes, hypertension, stroke, obesity, work physical activity, leisure physical activity, and country. * *p* < 0.001.

## Data Availability

The data that support the findings of this study are available via https://www.who.int/healthinfo/sage/en/ (accessed on 18 February 2021).

## Supplementary Materials

The following are available online at https://www.mdpi.com/2077-0383/10/6/1243/s1, Table S1: Prevalence of <-1 SD after adjustment for level of education and age for each cognitive performance test (overall and among those with mild cognitive impairment), Table S2: Prevalence of severity of difficulties with activities of daily living (overall and among those with mild cognitive impairment), Table S3: Association between active travel or covariates and mild cognitive impairment (outcome) estimated by multivariable logistic regression.
