# Supplementary material for: Active Travel and Mild Cognitive Impairment among Older Adults from Low- and Middle-Income Countries
Source: J Clin Med. 2021 Mar 17;10(6):1243. doi: 10.3390/jcm10061243 (PMC8002501; doi:10.3390/jcm10061243)
Supplement: Supplementary file 1 [file jcm-10-01243-s001.pdf]

APPENDIX

Table S1 Prevalence of <-1 SD after adjustment for level of education and age for each cognitive performance test (overall and among those with mild cognitive impairment)

Cognitive performance test	Overall (%)	MCI (%)
Immediate verbal recall	15.2	43.4
Digit span forward	16.2	41.4
Digit span backward	10.3	29.2
Animal naming task	12.5	24.3
Delayed verbal recall	16.3	47.8

Abbreviation: MCI Mild cognitive impairment

Table S2 Prevalence of severity of difficulties with activities of daily living (overall and among those with mild cognitive impairment)

Disability	Category	Overall (%)	MCI (%)
Difficulty getting dressed	None	90	85.7
	Mild	7.9	11.2
	Moderate	2.1	3.1
Difficulty eating	None	87.8	84.2
	Mild	8.9	12
	Moderate	3.3	3.8

Abbreviation: MCI Mild cognitive impairment

Table S3 Association between active travel or covariates and mild cognitive impairment (outcome) estimated by multivariable logistic regression

Characteristic		Overall		Age 50-64 years		Age ≥65 years	
		OR	95%CI	OR	95%CI	OR	95%CI
Active travel	High	1.00		1.00		1.00	
	Middle	0.97	[0.84,1.13]	0.95	[0.79,1.14]	1.04	[0.82,1.31]
	Low	1.33***	[1.14,1.54]	1.07	[0.89,1.30]	1.70***	[1.32,2.19]
Age (years)		1.03***	[1.02,1.04]	1.03***	[1.02,1.06]	1.05***	[1.04,1.07]
Sex	Female vs. Male	1.19	[0.99,1.43]	1.41**	[1.14,1.74]	1.01	[0.76,1.34]
Wealth	Poorest	1.00		1.00		1.00	
	Poorer	0.95	[0.81,1.13]	0.94	[0.76,1.16]	0.90	[0.69,1.18]
	Middle	1.06	[0.87,1.28]	0.90	[0.71,1.14]	1.21	[0.90,1.62]
	Richer	0.71***	[0.59,0.86]	0.70**	[0.55,0.89]	0.63***	[0.48,0.82]
	Richest	0.48***	[0.38,0.60]	0.39***	[0.29,0.52]	0.58**	[0.42,0.81]
Education (years)		1.00	[0.98,1.02]	0.99	[0.97,1.02]	1.00	[0.98,1.03]
Setting	Rural vs. Urban	0.59***	[0.50,0.69]	0.61***	[0.50,0.75]	0.61***	[0.48,0.77]
Alcohol consumption	Yes vs. No	1.02	[0.86,1.20]	0.98	[0.80,1.19]	1.16	[0.87,1.54]
Smoking	Never	1.00		1.00		1.00	
	Current	1.13	[0.95,1.34]	1.16	[0.96,1.41]	1.19	[0.88,1.61]
	Past	1.15	[0.93,1.43]	1.16	[0.85,1.59]	1.17	[0.85,1.61]
Sleep problems	Yes vs. No	2.02***	[1.61,2.53]	1.86***	[1.36,2.55]	2.16***	[1.60,2.92]
Anxiety	Yes vs. No	1.47*	[1.04,2.09]	1.64*	[1.00,2.69]	1.31	[0.82,2.08]
Depression	Yes vs. No	0.85	[0.62,1.15]	0.86	[0.59,1.28]	0.80	[0.51,1.27]
Diabetes	Yes vs. No	1.16	[0.92,1.45]	1.38	[0.98,1.94]	1.03	[0.78,1.37]
Hypertension	Yes vs. No	1.02	[0.92,1.14]	0.97	[0.85,1.10]	1.12	[0.93,1.35]
Stroke	Yes vs. No	1.94***	[1.51,2.49]	2.39***	[1.61,3.54]	1.56**	[1.11,2.19]
Obesity	Yes vs. No	1.34*	[1.07,1.68]	1.53**	[1.15,2.04]	1.16	[0.83,1.62]
Work physical activity	≤150 min per week	1.00		1.00		1.00	
	>150 min per week	1.11	[0.99,1.24]	1.29**	[1.10,1.52]	0.93	[0.79,1.10]
Leisure physical activity	≤150 min per week	1.00		1.00		1.00	
	>150 min per week	0.82	[0.65,1.03]	0.85	[0.64,1.13]	0.81	[0.57,1.15]

Abbreviation: OR Odds ratio; CI Confidence interval; Models are adjusted for all variables in the Table and country. * p<0.05, ** p<0.01, *** p<0.001
